# Representation of Tinnitus in the US Newspaper Media and in Facebook Pages: Cross-Sectional Analysis of Secondary Data

**DOI:** 10.2196/ijmr.9065

**Published:** 2018-05-08

**Authors:** Vinaya Manchaiah, Pierre Ratinaud, Gerhard Andersson

**Affiliations:** ^1^ Department of Speech and Hearing Sciences Lamar University Beaumont, TX United States; ^2^ LERASS Laboratory University of Toulouse Toulouse France; ^3^ Department of Behavioral Sciences and Learning Linköping University Linköping Sweden

**Keywords:** tinnitus, chronic condition, health communication, health information, media, news media, social media

## Abstract

**Background:**

When people with health conditions begin to manage their health issues, one important issue that emerges is the question as to what exactly do they do with the information that they have obtained through various sources (eg, news media, social media, health professionals, friends, and family). The information they gather helps form their opinions and, to some degree, influences their attitudes toward managing their condition.

**Objective:**

This study aimed to understand how tinnitus is represented in the US newspaper media and in Facebook pages (ie, social media) using text pattern analysis.

**Methods:**

This was a cross-sectional study based upon secondary analyses of publicly available data. The 2 datasets (ie, text corpuses) analyzed in this study were generated from US newspaper media during 1980-2017 (downloaded from the database US Major Dailies by ProQuest) and Facebook pages during 2010-2016. The text corpuses were analyzed using the Iramuteq software using cluster analysis and chi-square tests.

**Results:**

The newspaper dataset had 432 articles. The cluster analysis resulted in 5 clusters, which were named as follows: (1) brain stimulation (26.2%), (2) symptoms (13.5%), (3) coping (19.8%), (4) social support (24.2%), and (5) treatment innovation (16.4%). A time series analysis of clusters indicated a change in the pattern of information presented in newspaper media during 1980-2017 (eg, more emphasis on cluster 5, focusing on treatment inventions). The Facebook dataset had 1569 texts. The cluster analysis resulted in 7 clusters, which were named as: (1) diagnosis (21.9%), (2) cause (4.1%), (3) research and development (13.6%), (4) social support (18.8%), (5) challenges (11.1%), (6) symptoms (21.4%), and (7) coping (9.2%). A time series analysis of clusters indicated no change in information presented in Facebook pages on tinnitus during 2011-2016.

**Conclusions:**

The study highlights the specific aspects about tinnitus that the US newspaper media and Facebook pages focus on, as well as how these aspects change over time. These findings can help health care providers better understand the presuppositions that tinnitus patients may have. More importantly, the findings can help public health experts and health communication experts in tailoring health information about tinnitus to promote self-management, as well as assisting in appropriate choices of treatment for those living with tinnitus.

## Introduction

### Background

Tinnitus can be defined as the conscious perception of an auditory sensation in the absence of a corresponding external stimulus [1]. It is a common medical symptom, which can be debilitating to some individuals. Risk factors for tinnitus include hearing loss, use of ototoxic drugs, exposure to loud noise, and head injury. Various types of treatments are being offered to help tinnitus sufferers, although no effective curative drug treatment exists. Some main treatment and management approaches for tinnitus include sound therapy (ie, external noise to counteract the perception of tinnitus), hearing aids, and counseling [1,2]. However, cognitive behavioral therapy has the largest evidence base for the management of the distress associated with tinnitus [2].

### Self-Management of Chronic Conditions

Medical therapy alone is not sufficient to address the distress caused by various chronic symptoms and conditions [3], which can also be true for tinnitus. For this reason, there is a move toward developing self-management initiatives in which individuals with a health condition take responsibility and ownership in managing their health conditions and symptoms [4]. Self-management can facilitate acceptance, coping, learning to live well with chronic health conditions despite the adverse consequences of such conditions and symptoms, and more importantly can result in improved health outcomes [5]. However, when people with health conditions start to manage these health issues, a significant factor that determines how effectively they manage their issues involves the type and quality of information that they obtain through various sources (eg, news media, social media, health professionals, friends, and family). The information they gather helps form their opinions, and will also to some degree influence their attitudes toward managing their condition. Literature from health psychology, particularly the Self-Regulatory Model of Illness, provides a framework for understanding how individual symptoms and emotions during a health threat or diagnosis influence perception of illness and hence guide subsequent behavior [6,7]. An individual may be confronted with the problem of a potential illness through 2 channels, which include: (1) symptom perception and (2) social messages. The individual experiences and symptom perception can be influenced by the social messages they receive (ie, input from friends, family, and media). For this reason, health communication is an important element of facilitating self-management in chronic disease management from the public health viewpoint [8].

### Influence of Media on Health Behavior

The ubiquitous nature of mass media, in particular news media, makes it one of the most powerful sources of influence on specific issues (eg, climate change, political party), including health issues [9]. News media plays an important role in determining the content of our thoughts and opinions [10]. For example, the more coverage a specific topic receives on a particular news media, more likely are the people to take it into serious consideration. On the other hand, a topic that does not get much attention in the news media, although important, may not receive much public attention. Although society traditionally has relied heavily on the news media, in recent years, social media has radically transformed the way in which people communicate and gather information around the world [11]. Social media has changed the news business on a fundamental level [12]. For example, in social media content, providers are the first ones to report any given particular news, whereas editors and journalists are involved in content development and decision-making of what appears in the news media. People use social media to share and exchange perspectives and opinions about social, cultural, economic, and religious aspects; furthermore, there is a growing trend to also exchange information about health issues and concerns [13]. In keeping with this recent development, there is a critical need to better understand the influence of social media on health behavior and outcomes [14].

Although the media can help place specific health issues on a local and national agenda, there may be a discrepancy in what issues are covered and in their social and economic impact on society. For instance, we can assume that the issues more frequently raised in the media are those which are more likely to draw more attention and readership. Moreover, Hartz et al reports that there is a growing tension between media reporters, scientists, and health professionals [15]. This may be because of discrepancies in understanding specific health issues and also because of the importance placed on specific health issues by these key stakeholders [15].

There is some literature on how the deaf population is represented in the media [16,17]. For example, researchers have explored how and why hearing loss continues to be stigmatized through a study of media messages about hearing loss [16]. Another study focused on the positive and negative media framing toward political movement Deaf President Now [17]. Moreover, some recent literature also focuses on newspapers’ representation of workers with hearing loss [18] and the use of social media in the hearing aid community [19]. We recognize that both mass media and social media can have much influence on people’s knowledge and in the shaping of individual attitudes [14]. Although this is of importance, we were unable to find any studies focusing specifically on what information is presented about tinnitus in the news media, and also in social media.

This study aimed to understand how tinnitus is represented in the US newspaper media and in Facebook pages (ie, social media) using text pattern analysis.

## Methods

### Study Design

This study used a cross-sectional design based upon secondary analyses of publicly available data. The 2 datasets analyzed in this study were generated from US newspaper media and Facebook pages. The study did not require ethical approval as the data were gathered from publicly available sources. Only the publicly available Facebook pages (not personal pages) were included in the data extraction, thus maintaining the anonymity of the responses, and no personally identifiable information was included. Moreover, no individual dataset was discussed in the paper, again maintaining the anonymity of the data. Considering the minimal or no potential risk to individual participants, no ethical approval was required [20].

### Data Extraction

To develop the newspaper media text corpus (ie, large and structured set of texts), we first explored the databases with the newspaper collection available at Lamar University, United States. Major Dailies by ProQuest was the database that had the largest newspaper collection. We then searched for articles related to tinnitus in this database between the years 1980 and 2017 and downloaded the results as text corpus. A python script was written to convert the text corpus to a format that was needed for data analysis and to preserve the metadata (ie, newspaper name, year of publication).

A different python script was written to extract posts with Facebook pages dealing with tinnitus (during 2010-2016). In total, 20 Facebook pages with more than 100 likes were identified, and the postings were downloaded as a text corpus. It is important to note that the data extraction was limited to what data were available publicly (ie, newspaper data during 1980-2017 and Facebook posts during 2010-2016).

### Data Analysis

The text corpuses were analyzed using the Iramuteq software [21], which is an open source software. This software can perform various types of analyses on text data.

The text corpus is composed of multiple newspaper articles. The software treats each of these articles as text (ie, it’s the first unit). The first step of the analysis is to segment each article (ie, text) into smaller units called text segments (ie, each text is split into multiple text segments based on criteria of size and punctuation). The split of the text into segments decreases the granularity of the units and thus makes it possible to increase the precision of certain analysis, in particular the search of themes within the text corpus. The goal is to create segments of consistent size while trying to maintain the natural segmentation of texts marked by punctuation. The segmentation process is based on a cutoff criterion that weighs the segment size by punctuation. The procedure does not fully respect the parameterized segment size if a strong punctuation (eg, a period, a question mark, and an exclamation mark) is present within a 15% margin of the planned cutoff. In the next step, the text corpus is lemmatized (ie, words are sorted by grouping inflected or variant forms of the same word) to their simplest forms, which are called lemmas (ie, group of words in a single form). Moreover, the software makes distinctions between “full words” (eg, verbs, noun, adjectives, and adverbs) and “tool words” (eg, pronoun, determent, and useful verbs such as—to be and to have). This distinction is done so that only full words are included in the main analysis. These steps are necessary to convert the large corpus into a more manageable dataset that is essential for further analysis. To specifically analyze the text that is closely related to “tinnitus,” the text segments related to this object were extracted and new corpuses were formed. It is important to note that the expression “tinnitus” was inside each of the text segments extracted. The subcorpus with more directed text segments was used for all further analyses.

This was followed by cluster analysis made with the Reinert method used for textual data analysis [22-24]. The Reinert method has been used frequently in French media studies. This method facilitates the investigation of links between topics and the metadata associated with the text (eg, dates to study chronology). A recent comparison between the Reinert method and Latent Dirichlet Allocation showed that topics from the Iramuteq software that uses the Reinert method (ie, a divisive hierarchical clustering) are more accurate [25]. Although the traditional qualitative methods (eg, content analysis, thematic analysis) can provide in-depth understanding of the data, the automated text pattern analysis using the cluster analysis can provide a broader understanding of the data [22]. Automated analysis has the advantage of analyzing large amounts of data (ie, big data) as used in this study, whereas using the traditional qualitative methods for analyzing large quantities of data would not have been feasible.

**Figure 1:**
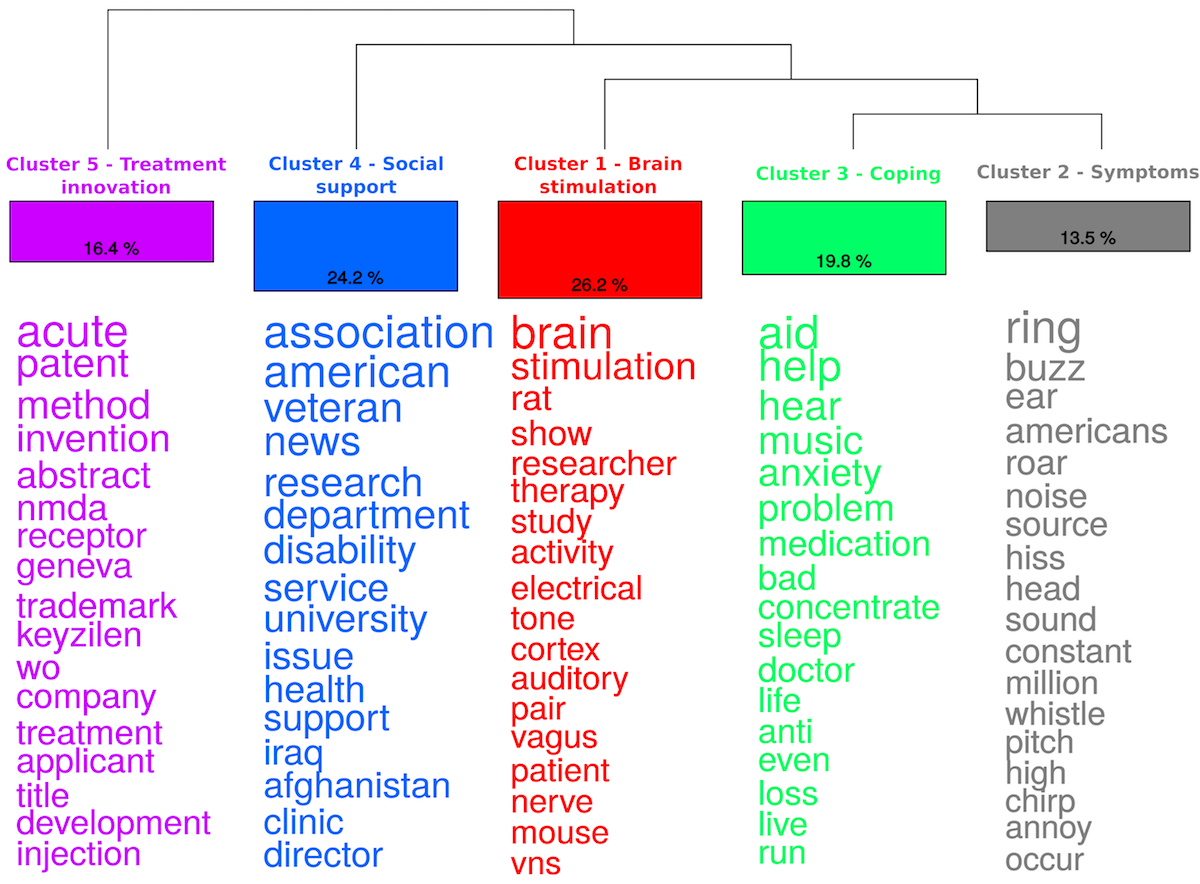
Dendrogram (ie, classification of clusters), size of clusters as percentage of the text segments, and overrepresented words in each cluster in tinnitus newspaper corpus. (Note: The words are ordered by chi-square value with words at the bottom having a lower value).

**Figure 2:**
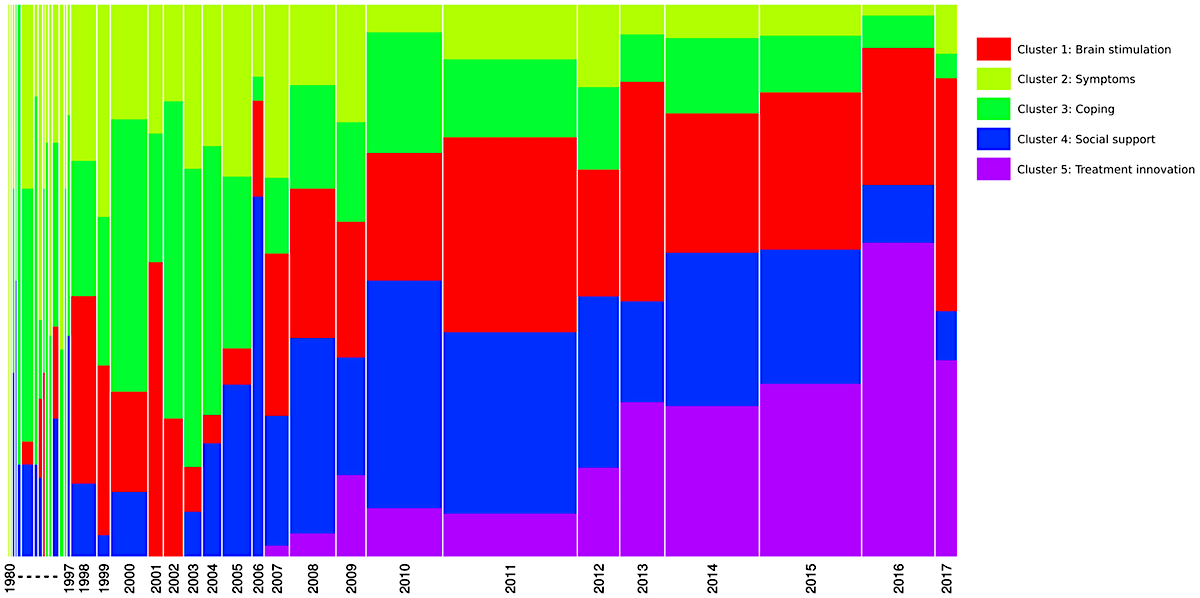
Chronological bar showing proportion of each cluster for each year in tinnitus US newspaper media corpus (Note: Width of the bar is proportional to the number of text segments each year, and the height of the clusters represents the frequency of text segments within clusters).

**Figure 3:**
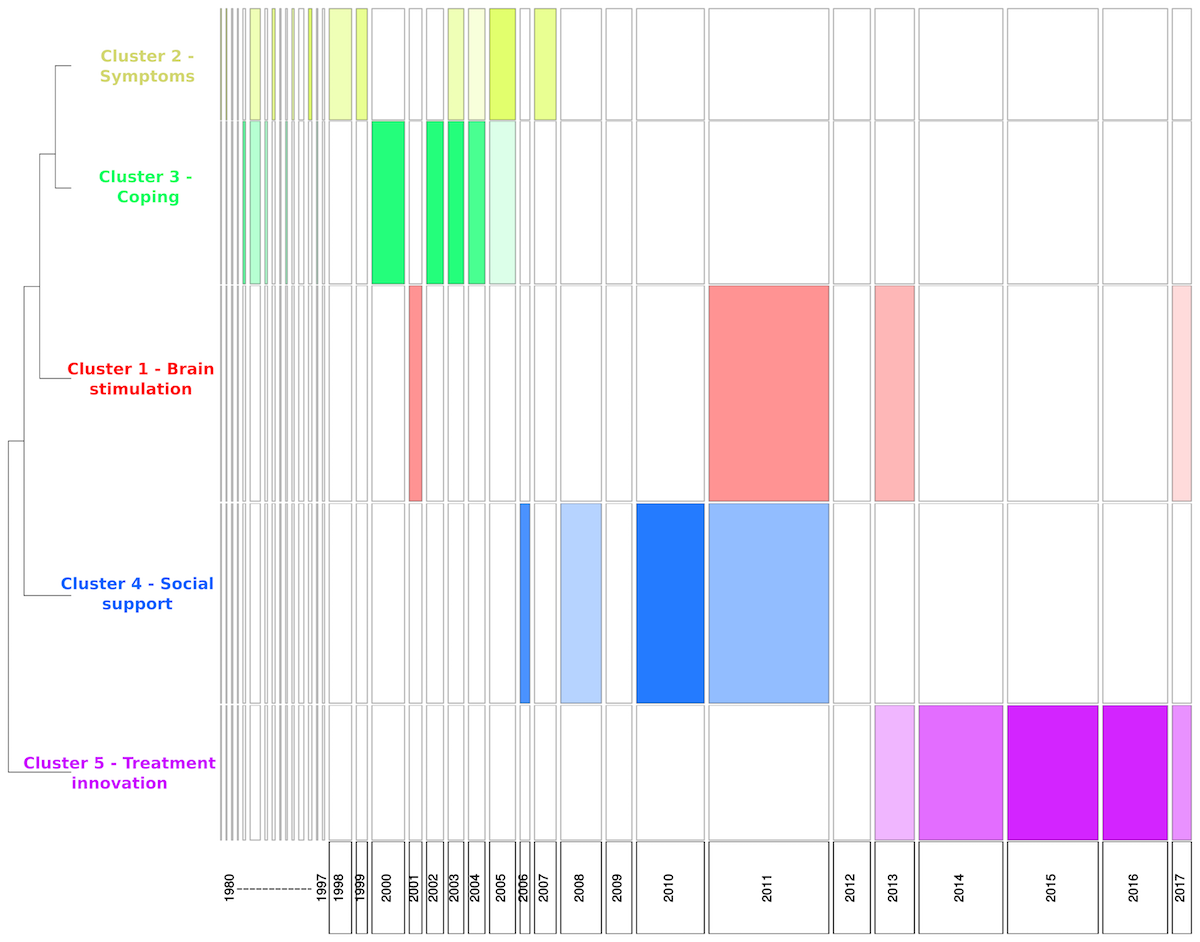
Chronological bar based on chi-square analysis showing proportion of each cluster for each year in tinnitus US newspaper media corpus (Note: Width of the bar is proportional to the number of text segments each year, and the height of the bar represents the size of clusters).

**Figure 4:**
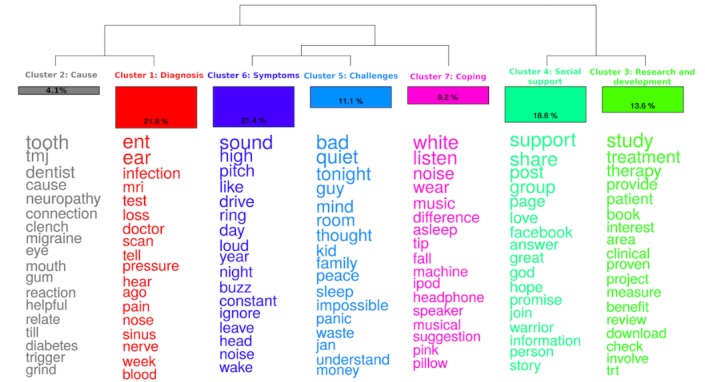
Dendrogram (ie, classification of clusters), size of clusters as percentage of the text segments, and overrepresented words in each cluster in tinnitus on Facebook pages corpus. (Note: The words are ordered by chi-square value with words at the bottom having a lower value).

**Figure 5:**
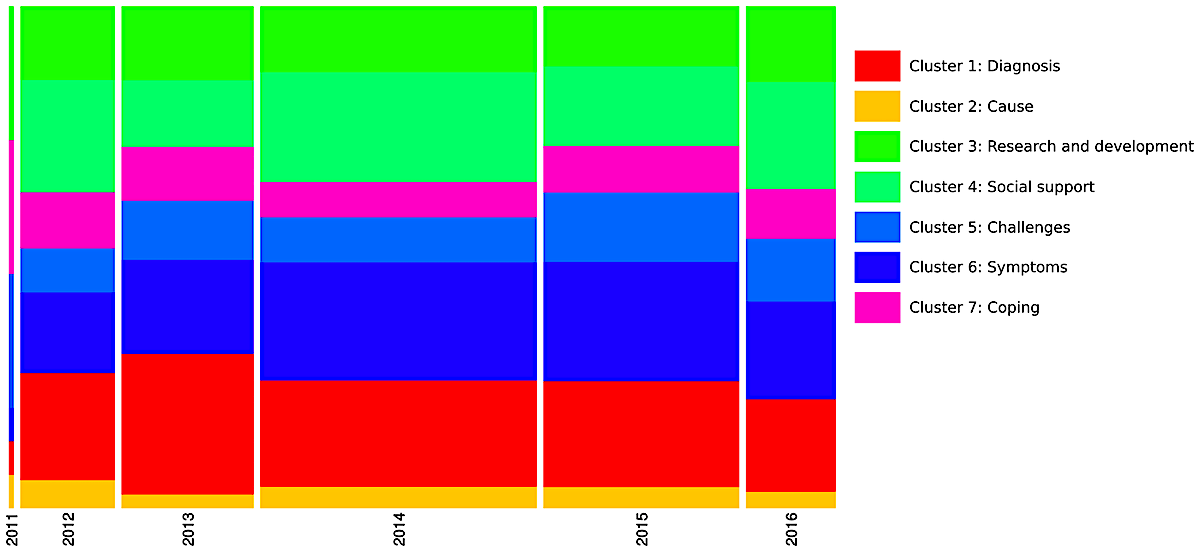
Chronological bar showing proportion of each cluster for each year on tinnitus Facebook pages corpus (Note: Width of the bar is proportional to the number of text segments each year, and the height of the clusters represents the frequency of text segments within clusters).

**Figure 6:**
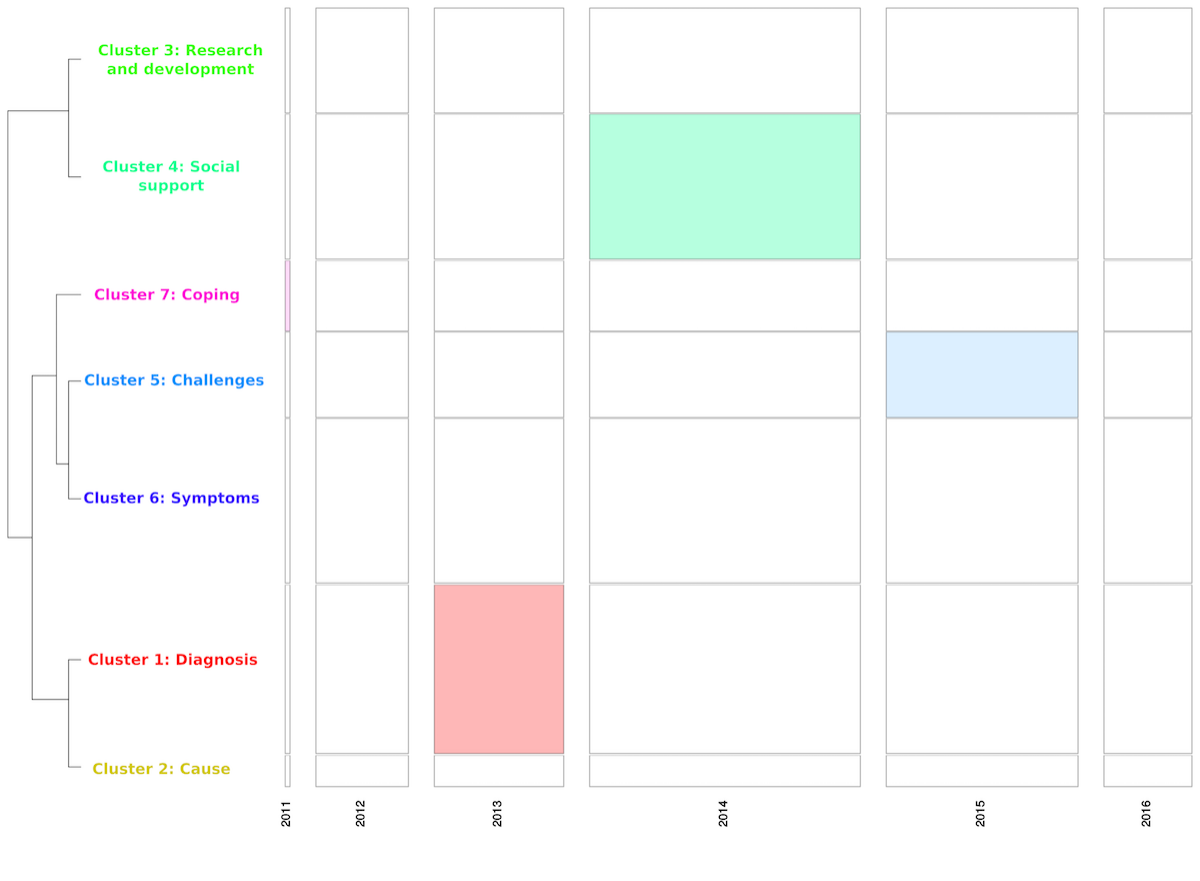
Chronological bar based on chi-square analysis showing the proportion of each cluster for each year on tinnitus Facebook pages corpus (Note: Width of the bar is proportional to the number of text segments each year, and the height of the bar represents the size of clusters).

In this analysis, the program initially builds a binary matrix with text segments in rows and full words in columns, and it then performs hierarchical divisive clustering based on a series of bipartitions made with correspondent analysis. At each step of the classification, the larger remaining cluster is cut into 2 parts by computing the information extracted while cutting after each line of the matrix along the first factor of the correspondent analysis. The remaining cut is the one that maximizes the information extracted. In a second step, each line is reversed from one cluster to the other. If this reversion increases the information extracted, it is kept. This step loops until no inversion increases the information extracted for the whole table. This cluster analysis groups the text segments based on co-occurrence of lemmas. Each of the clusters aim to be homogeneous (regrouping text segments with the common pattern of lemmas). Moreover, clusters have to be as heterogeneous as possible between them (pattern of lemmas between groups should be as different as possible). The results are presented in a dendrogram that characterizes the clustering. For each cluster, the program computes profiles of lemmas, which are overrepresented (ie, significantly in a higher proportion within the cluster when compared with the rest of the text corpus based on chi-square analysis). Finally, the same text corpus was subjected to a time series analysis using the metadata. For example, in this corpus, we analyzed how the patterns of clusters change over time (see Figures 1-6). Figures 2 and 5 present proportions of cluster for each year. In these figures, the width of the bar is proportional to the number of text segments each year (ie, the higher the width, the higher the number of text segments), and the height of the clusters (indicated in different colors) represents the frequency of text segments within clusters. Figures 3 and 6 present which clusters are significantly overrepresented (ie, based on chi-square analysis) in each year during 1990-2016. In this width of the bar, it is proportional to the number of text segments each year, and the height of the bar represents the size of clusters. For example, Figure 3 shows that cluster 5 is overrepresented during the years 2013-2017.

## Results

### Newspaper Data

#### Context Analysis

The initial analysis text corpus had 433 texts (ie, each article from a newspaper is considered a text), 9176 text segments (ie, each text is split into multiple text segments based on criteria of size and punctuation), and 309,524 occurrences or tokens (ie, number of words). After extraction of a text segment related to “tinnitus,” the text corps was reduced to 432 texts, 2173 text segments, and 79,684 occurrences or tokens.

Table 1 provides information about the newspaper source and the frequency of texts from each source. Most of the texts are from the US Fed News Services (30.79%), followed by The Washington Post (16.67%), Targeted News Service (13.19%), and the Chicago Tribune (10.19%). Table 2 provides information about the frequency of texts related to tinnitus over time. The frequency of texts published about tinnitus increases over time. The reason for this can be due to an increase in attention and publication of articles on tinnitus and/or the number of newspapers publishing articles about tinnitus. However, it is difficult to distinguish the relative contribution regarding these 2 reasons due to the increase in the frequency of texts related to tinnitus over time.

#### Content Analysis

Figure 1 provides results of cluster analysis on the newspaper data. The cluster analysis resulted in 5 main clusters that were named based on their characteristics. Cluster 1 was the largest cluster, which included 26.2% of texts, and mainly focused on aspects related to auditory and electrical stimulation in the brain as a cure for tinnitus, which was named as a *brain stimulation* cluster. Cluster 2 was the smallest cluster that included 13.5% of texts, mainly focusing on the symptoms of tinnitus such as buzzing, ringing, hiss, and was named as a *symptoms* cluster. Cluster 3 included 19.8% of texts and included aspects related to psychological problems (eg, anxiety), relaxation, and coping, and hence was named as a *coping* cluster. Cluster 4 was the second largest cluster and included 24.2% of the texts. This cluster had elements related to health and social support from government and charity organizations, and hence was named as a *social support* cluster. Cluster 5 included 16.4% of texts, and was focused on new and innovative treatment methods, and hence was named as a *treatment innovation* cluster. Table 3 provides examples of text segments that typically represent each of these 5 clusters.

**Table 1:** Frequency and percentage of articles containing at least one text-segment related to tinnitus in the subcorpus from different newspapers.

Newspaper	Frequency of articles, n (%)
Chicago Tribune	46 (11)
Farm Weekly	3 (0.7)
Investor’s Business daily	8 (1.9)
Journal of Commerce	1 (0.2)
Journal Record	2 (0.5)
Los Angeles Times	25 (5.8)
Marine Corps Times	1 (0.2)
Miami Daily Business Review	1 (0.2)
Missouri Lawyers Media	1 (0.2)
NASDAQ OMXs News Release Distribution Channel	30 (7.0)
New York Times	27 (6.3)
Roll Call	2 (0.4)
Targeted News Service	57 (13.1)
The Village Voice	2 (0.5)
The Washington Post	72 (16.7)
The Weekly Times	1 (0.2)
US Fed News Services	133 (30.8)
Wall Street Journal	20 (4.6)
Total	432 (100.0)

**Table 2:** Frequency and percentage of articles containing at least one text-segment related to tinnitus in the subcorpus based on timescales from different newspapers.

Year	Frequency of articles, n (%)
1980	1 (0.2)
1981	1 (0.2)
1985	2 (0.5)
1986	2 (0.5)
1987	3 (0.7)
1988	3 (0.7)
1989	3 (0.7)
1990	4 (0.9)
1991	1 (0.2)
1992	2 (0.5)
1993	3 (0.7)
1994	4 (0.9)
1995	3 (0.7)
1996	1 (0.2)
1997	1 (0.2)
1998	10 (2.3)
1999	7 (1.6)
2000	7 (1.6)
2001	3 (0.7)
2002	6 (1.4)
2003	7 (1.6)
2004	14 (3.2)
2005	43 (10.0)
2006	6 (1.4)
2007	21 (4.9)
2008	18 (4.2)
2009	14 (3.2)
2010	24 (5.6)
2011	46 (10.7)
2012	28 (6.5)
2013	20 (4.6)
2014	42 (9.7)
2015	41 (9.5)
2016	33 (7.7)
2017	8 (1.9)
Total	432 (100.0)

**Table 3:** Example of a text segment for each cluster in newspaper media text corpus.

Cluster	Example of a text segment
Cluster 1: *Brain stimulation*	Past research has *shown* that the *severity* of chronic *pain* and tinnitus is tied to the degree of *plasticity* in the *brain’s cortex* as a *previous study showed* that *repeatedly pairing sensory stimuli* with *electrical stimulation* of a *brain structure* called *nucleus* basalis *generates powerful* and *long-lasting* changes in *cortical* organization.
Cluster 2: *Symptoms*	*Commonly called ringing* in the *ears* but for *millions* of *people* who *suffer* tinnitus the *sound* that plagues them actually may be a *buzz hiss chirp* or *high-pitched whistle.*
Cluster 3: *Coping*	Playing *soft background music* or other *white noise* can be quite effective in *masking* the *noise* and short term use of *antidepressant medications* sometimes *helps* and often a *hearing aid helps* suppress tinnitus.
Cluster 4: *Social support*	A *senior member* of the *Senate Armed Services Committee* recently *received* the *senate* legislative *champion award* by the *American Tinnitus Association* for his dedicated *efforts* to include tinnitus *research* and *funding* to the *Annual National Defense* Authorization Act.
Cluster 5: *Treatment innovation*	As of *today* neither a *universal standard* of *care* for *acute* inner *ear* tinnitus nor a truly *proven* and *effective treatment method* is available about *Keyzilen TM* (AM-101). *Keyzilen TM* is a *small molecule N-methyl-D-aspartate receptor antagonist formulated* in a *biocompatible gel for intratympanic injection*.

**Table 4:** Example of a text segment for each cluster in Facebook pages text corpus.

Cluster	Example of a text segment
Cluster 1: *Diagnosis*	I went to an *ENT doctor 2 weeks ago.* He checked my *eustachian tubes* because my *ears* were *feeling* a bit *full* that’s all, no *ringing* no *pain* **.** He placed a probe up my *nose* but first *sprayed 2* pumps of lidocaine anesthetic.
Cluster 2: *Causes*	I have temporo mandibular *joint (TMJ)*, which has left me with very *limited jaw movement,* only *open mouth* to less than an inch between *teeth*. *I have* been referred to a *hospital,* but the waiting time is up to 2 years. Tinnitus can be caused by *TMJ*.
Cluster 3: *Research and development*	*Go Hearing*: We *provide tinnitus* and hearing *therapy* and *treatment* for Bay *area* and *Canada, providing* tinnitus lab pax 100 and tinnitus treater sound *conditioning programs,* which does not cost *thousands*. Affordable *effective programs* and *treatment, check* us out at www.go-hearing.com.
Cluster 4: *Social support*	Greetings, please *share* our *group page* in your *page* it is sometimes not about *money,* we want to *share* and *spread love* to all those who are *suffer* from tinnitus. Please do *support* us, we *need* your help.
Cluster 5: *Challenges*	It was *torture* and a *lot* of *bad thoughts* were running through my *mind*. I used *sleep* machines. We think it was from coming off a *drug* seroquel since *Jan* and Feb this year. I have the ringing 3-4 *times* a week.
Cluster 6: *Symptoms*	*Complete* cure to tinnitus: I had tinnitus in both *ears* for *15 years* with a *high-pitched* two-*tone sound.* The *noises* are *constant* and I have *learned* to *ignore* the *ringing.* Later another *sound* was *added* **.**
Cluster 7: *Coping*	Can a thyroid affect your tinnitus and my thyroid is coming out soon. I am hoping the hissing in my ear will subside a bit. I had to *sleep last night listening* to *white noise* for the first time in years. Usually I *listen* to the radio *playing music.*

#### Analysis of Trends Over Time

Figure 2 provides a chronological bar, and Figure 3 a chronological bar with chi-square for tinnitus newspaper data. These 2 figures provide information on how the information presented in newspaper media changes over time, and helps us understand media trends. For example, in Figure 2, it is evident that cluster 5 (ie, treatment innovation) was not discussed in newspapers before the year 2006, but the number of texts increased substantially from 2006 to 2017. The chi-square analysis (see Figure 3) shows that some clusters are more likely to occur on some timescale. For example, cluster 5 (ie, treatment innovation) was statistically significant and more frequent during the years 2013-2017, whereas the cluster 3 (ie, coping) was statistically significant and more frequent during the years 2000-2005. Overall, the time series analysis of clusters indicated changes in the pattern of information presented in newspaper media during the years 1980-2017.

### Facebook Pages Data

#### Context Analysis

Facebook pages text corpus had 1569 texts, 2747 text segments, and 78,218 occurrences or tokens. We have removed the URL and also texts that contained only the URL before analysis.

#### Content Analysis

Figure 4 provides results of cluster analysis on the Facebook pages data, which identify 7 main clusters. Cluster 1 (21.9%) was the largest and had words related to ear disorders and its diagnosis, and hence was named as *diagnosis* cluster. Cluster 2 (4.1%) was the smallest and was focused on causes and trigger factors of tinnitus (eg, tooth problems, neuropathy) and was named as the *cause* cluster. Cluster 3 included 13.6% texts focusing on studies that focused on tinnitus and was named as the *research and development* cluster. Cluster 4 (18.8%) had elements related to social support from various sources and was named as the *social support* cluster. Cluster 5 was named as challenges and had 11.1% of texts. Cluster 6 (21.4%) focused on symptoms of tinnitus such as buzzing, ringing, and hiss and was named as the *symptoms* cluster. Cluster 7 (9.2%) was focused on relaxation and coping, and hence was named as the *coping* cluster. Table 4 provides examples of text segments that typically represent each of these 7 clusters.

#### Analysis of Trends Over Time

Figure 5 provides a chronological bar and Figure 6 a chronological bar with chi-square for tinnitus Facebook pages data. As indicated in Figure 5, there is not much variation in what clusters are discussed in each year over time. The chi-square analysis (see Figure 6) shows that cluster 1 (ie, diagnosis), cluster 4 (ie, social support), and cluster 5 (ie, challenges) occurred more significantly during the years 2013, 2014, and 2015, respectively.

## Discussion

### Principal Findings

The media has played a prominent role in the dissemination of health information [9]. This study explores the way tinnitus is represented in the media, specifically in the US newspaper media and in Facebook pages using text pattern analysis. Overall, the study results suggest that newspaper media and Facebook pages offering information on tinnitus focus on various elements including symptoms, coping, diagnosis, social support, research and development, and treatment innovations.

The analysis of text corpus extracted from the US newspaper media suggested that the information disseminated via newspaper media focuses mainly on 2 elements: (1) new developments in treatments (ie, brain stimulation, treatment innovations) and (2) disease information (ie, symptoms, coping, and social support). It is interesting to note that there is a fairly equal spread of information among all of these elements. Moreover, the analysis trends over time regarding information indicated a change in patterns of information presented in the newspaper media during 1980-2017. For example, there is an increasing emphasis of information focusing on treatment inventions (cluster 5 in Figure 2), whereas the information on coping appears to be steady over the years (cluster 3 in Figure 2). We were unable to find previous studies on newspaper media on tinnitus to compare this study’s results. However, a recent study explored the Canadian newspaper representation of hearing-impaired workers [18]. The study results reveal that the media focuses on a wide range of issues (eg, hearing-impaired people taking action to find employment, and being determined to find success in sought after positions of employment).

The analysis of content in Facebook pages related to tinnitus in this study suggests that tinnitus sufferers use social media for various purposes, including gaining symptoms and diagnostic information, social support, learning to cope, and also to obtain information about research in this area. It is important to note that nearly half of the discussions in Facebook pages were related to diagnosis and symptoms (ie, 43.3%), suggesting that this platform is used by tinnitus sufferers for self-assessment of their condition. Facebook pages are fairly recent when compared with news media, and we explored Facebook pages information only during the years 2011-2016. Time series analysis showed no change in patterns of information during this time, unlike newspaper media. The results of this study relate well to previous studies on social media and other health conditions; for example, a recent study was conducted on the hearing aid community who used social media sources for advice and support, information sharing, and service-related information [19]. Analysis of information related to the level of usefulness of diabetes foot care-related Facebook groups showed a significant association with related posts [26]. Social media is also reported to be useful in creating indicators of the food environment that are associated with area-level mortality, health behaviors, and chronic conditions [27].

One would expect that the media would provide publicity for an intervention that is evidence-based. Although there are various treatments and/or management strategies available for tinnitus sufferers, psychological interventions such as cognitive behavioral therapy have the best evidence base for alleviating tinnitus distress [2]. However, it is surprising to see that there is a lack of publicity regarding psychological intervention, which becomes clear from the analysis of the information in the media. This is perhaps one of the reasons contributing to a relatively low uptake of evidence-based counseling interventions by tinnitus sufferers in the US population [28].

Overall, there is growing literature about the importance of health communication in chronic condition management, particularly concerning the role of mass media and social media in forming individuals’ opinions, and its bearing upon health behaviors. For this reason, it is important for health care professionals to be aware of the type of information that is being provided by the media on specific health conditions such as hearing loss and/or tinnitus.

### Study Implications

This study has several practical implications. As the media plays an important role in formulating people’s knowledge and opinions [14], it is important for health care professionals to understand the importance of media on health decision and behavior. This, we believe, may assist health care professionals in better understanding the presuppositions patients may have about various health aspects. It is suggested that when used wisely and prudently, social media can help promote individual and public health [29]. This study helps tinnitus researchers to understand the ways in which the public opinion about tinnitus is formed over time. Stakeholders including health care professionals, patient organizations, and public health specialists can use the media to ensure that appropriate and accurate information is being disseminated to promote positive health behavior and health outcomes [30]. However, the first step in this process is to understand the trends or patterns of information being presented in the media, as explored in this study.

### Study Limitations and Future Directions

This study is the first of its kind in the area of tinnitus. However, it has a few limitations. First, the text pattern analysis using software helps analyze big data, and although the analysis provides more of a macro view of the data, it may only provide a superficial understanding of its content. The automated data analysis may help us understand “what” aspects of tinnitus are represented in the media, rather than “how” tinnitus and these aspects are represented. For example, questions such as *Is brain stimulation portrayed positively or negatively in the media?*, or, *Is tinnitus portrayed as a curable condition?*, and finally, *How are the causes of tinnitus portrayed?* cannot be answered through this kind of analysis. Hence, in-depth qualitative analysis may be useful in future studies. Second, the newspaper data extracted from the ProQuest database may not be a true representation of all the US newspapers. Although ProQuest has some of the most widely circulated newspapers in the United States, using a different search engine may have potentially resulted in a different corpus and perhaps different results patterns. Furthermore, some selection bias may have occurred while choosing Facebook pages, as we only included 20 pages with 100 “likes.” Different criteria may have resulted in a different text corpus. This study mainly focuses on the patterns of information found in newspapers and in Facebook pages but not about how appropriate or accurate the information actually is. Hence, future studies should explore the appropriateness and accuracy of content. Moreover, future studies should aim to validate the results of automated text analysis using predictive validity methods, which confirms results by examining the data looking at the identified trends over time [31]. It would be interesting to study how the change over time may have occurred as a result of other metadata (eg, types of newspaper, focus of Facebook pages), which was not possible in this analysis due to limitations with software. Future studies should also consider studying the commonalities and differences among different types of media (eg, newspaper media, social media) using automated text analysis through more powerful software. Moreover, there is a great need to explore the role of the media on health behaviors, specifically in the tinnitus population.

### Conclusions

This study explored how tinnitus is represented in the media, specifically in the US newspaper media and in Facebook pages. The information in the newspapers regarding tinnitus was mainly about brain stimulation, symptoms, coping, social support, and treatment innovations. Time series analysis showed that there were some changes in the patterns of information presented in newspaper media during 1990-2017. The information in Facebook pages about tinnitus was mainly related to diagnosis, cause, research and development, social support, challenges, symptoms, and coping. However, no changes in the patterns of information presented in Facebook pages between 2011 and 2016 were noted. These findings can help clinicians to better understand presuppositions tinnitus patients may have in regard to their condition. In addition, the findings can also be of interest to public health and health communication experts, allowing them to tailor health information about tinnitus to promote self-management and appropriate treatment choices for tinnitus sufferers.
